# Borderline personality traits mediate the relationship between negative life events and nonsuicidal self-injury in a clinical sample with youth depression

**DOI:** 10.1186/s12888-024-05821-0

**Published:** 2024-05-16

**Authors:** Wangni Chen, Ting Yuan, Yuwen Pan, Yarong Ma, Bin Sun, Min Yu, Xiaoming Lin, Hongbo He, Jie Zhang

**Affiliations:** 1 Department of Psychosomatic Medicine, The Affiliated Brain Hospital of Guangzhou Medical University, 36 Mingxin Road, Liwan District, Guangzhou, Guangdong 510370 China; 2 Key Laboratory of Neurogenetics and Channelopathies of Guangdong Province and the Ministry of Education of China, Guangzhou Medical University, Guangzhou, China; 3 Guangzhou Medical University, Guangzhou, 510000 China; 4 Guangdong Mental Health Center, Guangdong Provincial People’s Hospital (Guangdong Academy of Medical Sciences, Southern Medical University, Guangzhou, Guangdong 519041 China

**Keywords:** Depression, Borderline personality traits, Nonsuicidal self-injury, Adolescent negative life events, Youth

## Abstract

**Background:**

Borderline personality traits play a significant role in nonsuicidal self-injury (NSSI), particularly in depressed youths. NSSI is also highly correlated with negative life events. This research aimed to explore the connections between negative life events, borderline personality traits, and NSSI.

**Methods:**

The study included 338 depressed youth aged 13 to 25 years. Self-reported measures and clinical interviews were utilized to evaluate the depressive symptoms, borderline personality traits, negative life events, and NSSI behaviours of these participants. Identifying variables linked to NSSI was the aim of our analysis, and we also conducted a mediation analysis to look into the influence of borderline traits on the connection between negative life events and NSSI.

**Results:**

Of the 338 depressed youth, approximately 59.47% (201/338) displayed NSSI, which was associated with greater clinical severity. Borderline traits had an independent influence on NSSI and it partially explained the connection between negative life events and NSSI, even when accounting for depression symptoms. Depressed youth who were more vulnerable to NSSI behaviours often experienced negative life events such as interpersonal relationships, academic pressure, being punished, and loss.

**Conclusions:**

Our research suggests that depressed youth who experience more negative life events are more likely to experience NSSI, and negative life events indirectly influence nonsuicidal self-injury through borderline personality traits. Implementing interventions focused on mitigating borderline symptoms could be a promising therapeutic approach for addressing NSSI in young people.

**Supplementary Information:**

The online version contains supplementary material available at 10.1186/s12888-024-05821-0.

## Introduction

Nonsuicidal self-injury (NSSI) occurs when an individual deliberately causes damage to bodily tissues without the explicit intention of terminating their own life [1]. Adolescence is a critical and susceptible period in which NSSI can manifest [2]. Among the global population of children and adolescents, approximately 19.5% display nonsuicidal self-injury behaviours [3]. Previous data have highlighted that adolescents and young adults have a higher incidence of NSSI than adults (pooled prevalence in adolescents, young adults and adults are 17.2%, 13.4% and 5.5% ) [4]. Recent researches have suggested that bullying, stressful events, personality traits, and mental disorders play critical roles in both the initiation and perpetuation of NSSI during this critical period of development [5–7]. Moreover, research has shown that NSSI and suicidal behaviours are interrelated. Suicidal intentions and actions are potentially linked to the late development and persistence of NSSI [8]. Acquiring a thorough awareness of the contributing factors associated with the onset of NSSI, particularly in depressed youth, is of utmost importance. This will help in maximizing prevention efforts and further treatment.

Personality traits are considered most strongly associated with NSSI behaviour, especially in patients with borderline traits [6, 9]. Borderline personality disorder (BPD) is a widely recognized psychiatric condition characterized by the ubiquitous destabilization of interpersonal relationships, self-inflicted harm, impulsive behaviours, and emotional fluctuations [10]. NSSI occurs in approximately 90% of adult BPD patients, with two-thirds reporting an incident prior to reaching the age of 18 [6]. NSSI may be considered as a detectable risk indicator for the early identification of BPD symptoms in young people [6]. Additionally, core features of BPD, including interpersonal and emotional instability [11], presage and interact with NSSI. Thus, it is possible to consider the fundamental symptoms of BPD as contributing to the recurrence of NSSI [12]. A previous study revealed that BPD traits predict persistent NSSI for twelve months in individuals with a minimum of one NSSI [13]. Consequently, there is a prevailing assumption that BPD characteristics come before and exert an impact on NSSIs [6]. Given that NSSI is a distinctive feature of BPD, it is essential to thoroughly elucidate the association between these two entities.

The various stressful stimuli that people encounter in their social lives are known as negative life events. (for example, “having conflicts with friends,” “failing in tests,” or “losing relatives and friends”) [14]. Individuals diagnosed with BPD encounter adverse environmental life events at a greater frequency than individuals in the control group [15]. Adverse childhood life events may modulate gene expression, contribute to unstable personality traits, and increase the likelihood of encountering extraordinary life occurrences [16–18]. Life-threatening traumatic experiences, including sexual or physical abuse and disturbed relationships, have been found to be significant contributors to BPD development [14, 19].

According to the stress generation hypothesis [20], being exposed to higher levels of life stressors enhances the likelihood of experiencing subsequent adverse mental health outcomes, particularly for individuals with vulnerabilities. Having difficulty coping with negative life events is also thought to be connected with the occurrence of depression [21] and could increase the likelihood of suicidal thoughts and behaviours, especially for adolescents [22]. Additionally, life stressors have received considerable empirical attention because they are a significant contributing factor to the occurrence of NSSI [23]. When individuals encounter specific stressors, they employ various coping mechanisms. However, those with a predisposition to emotional regulation challenges, such as individuals exhibiting borderline personality traits, tend to employ NSSI as a coping strategy. This behaviour can alleviate negative emotions and provide a sense of gratification [24].

While multiple studies have illustrated the impact of adverse life events on NSSI behaviours in depressed youth, the exploration of the mediating mechanisms underlying this association has been limited to a few studies. In a teenage nonclinical sample, a previous study revealed that emotional symptoms partially explain the connection between adverse life events and NSSI [25]. Similarly, negative life events were also found to be connected with anxiety and further increased NSSI in another study [26]. Notably, these studies have concentrated solely on the connection between emotional symptoms within the context of adverse life events and NSSI. Nevertheless, despite the robust correlation between NSSI and borderline personality traits, research on the potential mediating function of borderline personality traits is limited.

The main purpose of this research was to investigate the factors associated with affecting NSSI in a clinical group of depressed teenagers. Our hypothesis suggests that an elevated occurrence of adverse life events and the presence of borderline characteristics are strongly associated with an increasing frequency of NSSI in depressed youth. Simultaneously, we delved into the potential mediating function of borderline traits in the association between adverse life experiences and NSSI behaviours. Based on prior research [25, 26], we assumed that the connection between negative life events and NSSI would be mediated by the presence of borderline traits. Additionally, given that two-thirds of depressive patients with borderline traits exhibit suicidal behaviour before age 18 and that ageing is strongly associated with personality refinement [6], we conducted subgroup analyses of depressed youth at 18-year intervals to explore whether there were any differences in terms of negative life events and NSSI among minors and early adults.

## Method

### Participants

Individuals in this cross-sectional study were those who visited the Brain Hospital of Guangzhou Medical University from January 2022 to May 2023. The inclusion criteria were age 13 to 25 years, diagnosis of either unipolar or bipolar depression according to the The International Statistical Classification of Diseases and Related Health Problems 10th Revision (ICD-10) diagnostic criteria, and written informed consent forms must be signed by both parents and patients. The exclusion criterion was the inability to complete the questionnaire due to comprehension difficulties or intellectual deficits. Additionally, individuals who reported self-harm with suicidal intent were excluded from the research if they were in the NSSI group.

A total of 391 patients were ultimately recruited. Fifty-three patients were excluded (those who reported self-harm with suicidal intent), and 338 patients composed the final study sample. Subsequently, the patients were separated into two distinct cohorts based on their previous NSSI behaviours (NSSI group vs. non-NSSI group: 201 vs. 137). In Table 1, demographic characteristics are detailed. Our study was approved by the Ethical Committee of the Affiliated Brain Hospital of Guangzhou Medical University (ID number: 2022-031). Informed consent was obtained from all subjects and/or their legal guardians.

**Table 1:** Sociodemographic characteristics of the sample

Characteristic	No. (%)			t/Z/X^2^	*P* value
	Total (N = 338)	NSSI (N = 201)	Non-NSSI (N = 137)		
Age, Mean (SD)	19.06 (3.23)	18.40 (3.19)	20.03 (3.06)	4.679	< 0.001
Female	252 (74.6)	167 (83.1)	85 (62.0)	19.013	< 0.001
Education				18.755	< 0.001
Middle school	10 (3.0)	4 (2.0)	6 (4.4)		
Junior high school	60 (17.8)	47 (23.4)	13 (9.5)		
Senior high School or secondary school	110 (32.5)	72 (35.8)	38 (27.7)		
University or above	158 (46.7)	78 (38.8)	80 (58.4)		
Marriage				3.621	0.164
Single	248 (73.4)	143 (71.1)	105 (76.6)		
In love	75 (22.2)	51 (25.4)	24 (17.5)		
Married	15 (4.4)	7 (3.5)	8 (5.8)		
Work				7.469	0.048
Full-time job	56 (16.6)	27 (13.4)	29 (21.2)		
Part-time job	4 (1.2)	4 (2.0)	0 (0.0)		
Students	255 (75.4)	159 (79.1)	96 (70.1)		
Unemployment	23 (6.8)	11 (5.5)	12 (8.8)		
Family income per month				3.399	0.334
<3000	21 (6.2)	15 (7.5)	6 (4.4)		
3000–5000	90 (26.6)	55 (27.4)	35 (25.5)		
5000–10,000	109 (32.2)	58 (28.9)	51 (37.2)		
>10,000	118 (34.9)	73 (36.3)	45 (32.8)		
History of suicide attempts				20.068	< 0.001
None	206 (60.9)	103 (51.2)	103 (75.2)		
1–2 times	120 (35.5)	88 (43.8)	32 (23.4)		
≥ 3 times	12 (3.6)	10 (5.0)	2 (1.5)		

### Procedure

Initially, researchers with clinical expertise and backgrounds in psychology collected demographic and pertinent clinical information from the participants. All the researchers passed the scale consistency assessments. Then, the participants were asked to fill out self-report questionnaires using an online platform.

### Measures

#### 23-item borderline symptom list (BSL‐23)

Utilizing a 23-item self-report scale, the BSL-23 evaluates the subjectivity of the previous week’s BPD symptom severity [27, 28]. Each item is accompanied by a range from 0 to 4 based on the extent of suffering, where larger cumulative BSL–23 scores signify greater degrees of borderline traits. Excellent internal consistency was observed for the BSL-23 in this investigation (Cronbach’s α = 0.964).

#### Adolescent self-rating life events checklist (ASLEC)

The 26 items on the Adolescent Self-Rating Life Events CheckList [29] constitute a five-point Likert scale (1 = no influence, 5 = severely influence) that inquires about the psychological states of subjects during negative life events that occurred within the previous year. Subjects with higher scores demonstrate a stronger impact of adverse life events. In this investigation, the Adolescent Self-Rating Life Events CheckList exhibited satisfactory internal consistency (Cronbach’s α = 0.857).

#### The 17-item hamilton depression rating scale (HAMD-17)

The 17-item Hamilton Depression Rating Scale [30] is assessed using a 5-point Likert scale that spans from 0 to 4. The cumulative score on this scale varies from 0 to 68. Greater scores are indicative of an increased prevalence of depressive symptoms. In this study, the HAMD-17 scale exhibited excellent internal consistency (Cronbach’s α = 0.836).

#### The brief non-suicidal self-injury assessment tool

The Brief Non-Suicidal Self-Injury Assessment Tool [31, 32] consists of five sections covering (a) questions related to self-injurious behaviours, (b) their purposes, (c) how often and when they occurred (including the age of initiation and cessation), (d) the locations of wounds, and (e) the initial motivations behind these behaviours. To assess NSSI, participants were queried with the question: “Have you ever intentionally used any of the following methods to harm yourself?” This query was followed by a list of 16 NSSI behaviours, as well as an “other” option. Additionally, participants were asked about the reasons behind their intentional self-harm and whether practising or attempting suicide was the primary motive. If the response was “Yes” to the latter, they were then omitted from the NSSI group.

### Statistical analyses

We used IBM SPSS Statistics (version 25.0; SPSS Inc., Chicago, United States) to investigate disparities between groups (NSSI vs. non-NSSI), and we performed an independent samples t test or Mann-Whitney U test to test for continuous variables, based on whether the data were normally distributed. Categorical variables were analysed using Pearson’s chi-square tests. Additionally, we performed multivariate logistic regression to investigate potential dangers linked to NSSI behaviour.

#### Logistic regression analysis

To represent whether NSSI was present or not in the prior year, the dependent variable was dichotomized. Age, sex, education level, lifetime suicide attempts, borderline traits (BSL-23), depressive symptoms (HAMD-17), and adolescent negative life events (ASLEC) were among the explanatory variables considered. Initial analyses involved conducting univariate logistic regressions for all predictors, which resulted in the calculation of odds ratios (ORs) accompanied by 95% confidence intervals (CIs). Subsequently, in the multivariate logistic regression model, we incorporated the variables that showed statistical significance in the univariate analyses. To determine the final model, an enter selection method was employed. To avoid type I errors, multiple-comparison correction was performed using Bonferroni correction [33] before performing the multiple regression analysis, in order to determine whether multicollinearity was present, the variation inflation factor (VIF) was computed for every predictor. Significantly, every VIF value was observed to be less than 3, suggesting the absence of multicollinearity concerns [34].

#### Mediation analysis

**Fig. 1:**
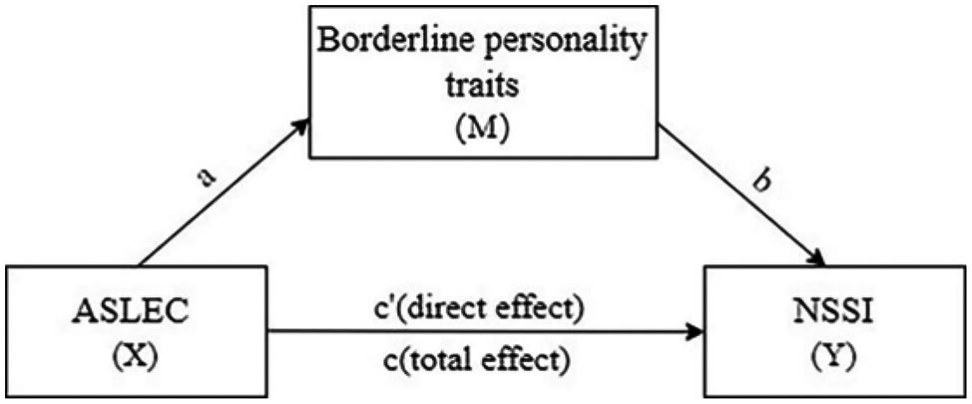
Graphical representation of a mediation model on the influence of borderline traits on the connection between negative life events (ASLEC) and non-suicidal self-injury (NSSI)

The PROCESS program (Model 4) developed by Hayes [35] was used to perform the mediation analysis to assess the potential mediating role of borderline traits (BSL-23) in the link between adverse life events during adolescence and nonsuicidal self-injury. Then we established a mediation model, as displayed in Fig. 1. Parameter estimation was carried out using the bias-corrected nonparametric percentile bootstrap method. Overall, 5,000 bootstrapped samples were employed to compute indirect effects and establish 95% confidence intervals, to assess the significance of these indirect effects. If the 95% confidence interval included zero, both the indirect effects and coefficients were deemed insignificant. Furthermore, sex, history of suicide attempts, and severity of depression were adjusted for as covariates in the models. Before estimating the indirect effect, standardization was applied to the coefficients [36]. Additionally, given that two-thirds of depressed patients with borderline traits had engaged in suicidal behaviour before turning 18, participants were stratified based on an age interval of 18 years. This stratification allowed us to perform the mediation analysis again to detect potential differences in the correlation between negative life events and nonsuicidal self-injury behaviours among minors and young adults.

## Results

### Sample characteristics

Among the 338 patients with depressive disorders, approximately 59.47% (201/338) exhibited NSSI. Those exhibiting NSSI tendencies were more inclined to be female (83.1%), younger (with a mean age of 18.40 year, SD 3.19), have a junior high school or senior high school degree (X^2^ = 18.755, *P* < 0.001) and have attempted suicide in the past (X^2^ = 20.068, *P* < 0.001) (Table 1).

In contrast, individuals displaying NSSI behaviours exhibited more pronounced depressive symptoms, a greater occurrence of negative life events, and more evident borderline traits than did those without NSSI behaviours (Table 2). Specifically, among individuals with NSSI behaviours, the factors of anxiety/somatization, cognitive impairment and hindrance according to the HAMD-17 scale were notably elevated. Additionally, the negative life events experienced by young people were more closely associated with interpersonal relationships, academic pressure, being punished, loss, health-related adjustment, and other factors (Table 2).

**Table 2:** Between-group differences in borderline traits, depression symptom and negative life events

Clinical symptom	Total (N = 338)	NSSI (N = 201)	Non-NSSI (N = 137)	t/Z	*P* value
Borderline traits (BSL-23)					
BSL-23 total, Mean ± SD	44.27 ± 23.42	50.66 ± 21.76	34.89 ± 22.67	-6.428	< 0.001
Depression symptom(HAMD-17)					
Anxiety/somatization, Mean ± SD	6.65 ± 3.34	6.97 ± 3.06	6.19 ± 3.67	-2.050	0.041
Cognitive impairment, Mean ± SD	3.87 ± 2.20	4.39 ± 2.13	3.11 ± 2.09	-5.484	< 0.001
Weight, M (Q_1_, Q_3_)	0.00 (0.00–1.00)	0.00 (0.00–1.00)	0.00 (0.00–1.00)	-0.237	0.813
Hindrance, Mean ± SD	4.79 ± 2.16	4.99 ± 2.16	4.49 ± 2.13	-2.105	0.036
Sleep disturbance, Mean ± SD	2.62 ± 1.93	2.73 ± 1.96	2.46 ± 1.89	-1.270	0.205
HAMD-17 total, Mean ± SD	18.43 ± 8.06	19.60 ± 7.71	16.70 ± 8.29	-3.296	0.001
Negative life events (ASLEC)					
Interpersonal relationship, Mean ± SD	12.68 ± 4.71	13.97 ± 4.70	10.79 ± 4.07	-6.617	< 0.001
Academic pressure, Mean ± SD	11.41 ± 4.11	11.96 ± 4.28	10.61 ± 3.73	-2.978	0.003
Being punished, M (Q_1_, Q_3_)	11.00 (9.00–15.00)	12.00 (10.00–17.00)	10.00 (8.00–12.00)	-5.620	< 0.001
Loss, M (Q_1_, Q_3_)	4.00 (3.00–6.00)	4.00 (3.00–6.00)	4.00 (3.00–5.00)	-2.621	0.009
Health-related adjustment, Mean ± SD	8.33 ± 2.72	8.64 ± 2.90	7.88 ± 2.38	-2.641	0.009
Others, Mean ± SD	8.17 ± 2.93	8.71 ± 2.96	7.39 ± 2.72	-4.175	< 0.001
ASLEC total, Mean ± SD	56.75 ± 16.17	60.73 ± 16.49	50.92 ± 13.80	-5.925	< 0.001

*Note* BSL-23 = 23-Item Borderline Symptom List. HAMD-17 = the17-item Hamilton Depression Rating Scale (Anxiety/somatization, cognitive impairment, weight, hindrance and sleep disturbance are factors of HAMD-17). ASLEC = Adolescent Self-Rating Life Events CheckList (Interpersonal relationship, academic pressure, being punished, loss, health-related adjustment, and others are factors of ASLEC )

### Logistic regression analysis

Univariate logistic regression analyses revealed significant predictive power for engaging in NSSI, including participants’ gender, age, education level, history of suicide attempts, depressive symptoms (measured by the HAMD-17), borderline traits (measured by the BSL-23), and negative life events experienced during adolescence (measured by the ASLEC) (refer to Table 3). However, after controlling for all potential confounding variables in the multivariate logistic regression analysis, the following factors were considered to be important in predicting NSSI: being female (OR = 2.87, 95% CI 1.58–5.23, *P* < 0.001), having a history of suicide attempts (*P* = 0.008), displaying borderline traits (OR = 1.03, 95% CI 1.01–1.05, *P* < 0.001), and experiencing negative life events, particularly those related to interpersonal relationships (OR = 1.10, 95% CI 1.01–1.19, *P* = 0.022). Notably, depressive symptoms did not significantly contribute to this model (Table 3).

**Table 3:** Univariate and multivariate logistic regression analyses examining predictors of NSSI

Non-suicidal Self-injury	Univariate Analyses			Multivariate Analyses		
	OR (95%CI)	*P* value	*P* value adjusted	OR (95%CI)	*P* value	*P* value adjusted
Age	0.85 (0.79–0.91)	< 0.001	< 0.001	0.93 (0.83–1.05)	0.245	1.000
Sex						
Female	3.00 (1.81–4.98)	< 0.001	< 0.001	2.87 (1.58–5.23)	0.001	0.017
Education		< 0.001	0.012		0.173	1.000
Junior high school	1.91 (0.92–3.96)	0.082	1.000	5.65 (1.15–27.83)	0.033	0.561
Senior high School or secondary school	0.51 (0.31–0.85)	0.009	0.261	4.58 (1.02–20.54)	0.047	0.799
University or above	0.35 (0.09–1.32)	0.122	1.000	3.41 (0.74–15.79)	0.116	1.000
Marriage		0.168	1.000			
In love	1.56 (0.90–2.70)	0.111	1.000			
Married	0.64 (0.23–1.83)	0.407	1.000			
Work		0.172	1.000			
Part-time job	0.00 (0.00-Inf)	0.999	1.000			
Students	1.78(0.99–3.18)	0.052	1.000			
Unemployment	0.99(0.37–2.60)	0.975	1.000			
Family income per month		0.340	1.000			
3000–5000	0.63(0.22–1.77)	0.380	1.000			
5000–10,000	0.46 (0.16–1.26)	0.130	1.000			
>10,000	0.65 (0.24–1.80)	0.405	1.000			
History of suicide attempts		< 0.001	0.002		0.008	0.136
1–2 times	2.75 (1.69–4.48)	< 0.001	0.001	2.40 (1.36–4.26)	0.003	0.042
≥3 times	5.00 (1.07–23.38)	0.041	1.000	3.19 (0.44–23.10)	0.252	1.000
BSL-23	1.03 (1.02–1.04)	< 0.001	< 0.001	1.03 (1.01–1.05)	< 0.001	0.003
HAMD-17	1.05 (1.02–1.08)	0.001	0.029	0.98 (0.94–1.02)	0.304	1.000
ASLEC	1.04 (1.03–1.06)	< 0.001	< 0.001			
Interpersonal relationship	1.17 (1.11–1.24)	< 0.001	< 0.001	1.10 (1.01–1.19)	0.022	0.374
academic pressure	1.09 (1.03–1.15)	0.004	0.116	0.96 (0.88–1.04)	0.281	1.000
Being punished	1.16 (1.10–1.23)	< 0.001	< 0.001	1.09 (0.99–1.19)	0.086	1.000
Loss	1.11 (1.02–1.21)	0.019	0.551	1.03 (0.92–1.16)	0.573	1.000
Health-related adjustment	1.11 (1.02–1.21)	0.012	0.348	0.96 (0.84–1.11)	0.615	1.000
Others	1.18 (1.09–1.28)	< 0.001	0.002	0.91 (0.80–1.04)	0.164	1.000

*Note* OR = Odds Ratio. BSL-23 = 23-Item Borderline Symptom List. HAMD-17 = the17-item Hamilton Depression Rating Scale. ASLEC = Adolescent Self-Rating Life Events CheckList.

P value adjusted: using the Bonferroni correction

### Mediation analysis

When borderline features were incorporated into the regression analysis, the effect of specific aspects of negative life events on NSSI vanished. This study aimed to investigate the potential explanatory role of borderline traits in the connection between negative life events and NSSI, and seven mediation analyses were performed in total. We sought to determine whether the connection between more frequent negative life events and NSSI could be clarified by the positive correlation of ASLEC scores with borderline traits. To account for the significant predictive roles of gender and history of suicide attempts, as well as the strong association between depression severity and NSSI, covariates were incorporated into the analysis for these variables.

The results illustrating the mediating effect between negative life events and NSSI through borderline traits are presented in Table 4 and Supplemental Fig. 1.An additional movie file shows this in more detail [see Additional file 1].

**Table 4:** Results of mediating effect for borderline traits between adolescent negative life events and NNSI.

Paths	Bootstrap effects								
	Total effect	LLCI	ULCI	Indirect effect	LLCI	ULCI	Direct effect	LLCI	ULCI
Total negative life events →Borderline traits→non-suicidal self-injury	0.5994	0.312	0.8868	0.1896	0.0764	0.3286	0.4178	0.1124	0.7232
Interpersonal relationship→Borderline traits→non-suicidal self-injury	0.6798	0.4009	0.9588	0.1655	0.0666	0.2869	0.5334	0.2401	0.8267
Academic pressure→Borderline traits→non-suicidal self-injury	0.2586	0.0071	0.5101	0.1185	0.0473	0.2354	0.1412	-0.1254	0.4078
Being punished→Borderline traits→non-suicidal self-injury	0.5620	0.2793	0.8446	0.1411	0.0594	0.2568	0.4293	0.1336	0.7142
Loss→Borderline traits→non-suicidal self-injury	0.2546	0.0026	0.5065	0.083	0.0177	0.184	0.1927	-0.071	0.4563
Health-related adjustment→Borderline traits→non-suicidal self-injury	0.2220	-0.0495	0.4935	0.1504	0.0625	0.2693	0.0693	-0.221	0.3598
Others→Borderline traits→non-suicidal self-injury	0.3347	0.0711	0.5984	0.2305	0.1073	0.3986	0.0975	-0.1944	0.3894

LLCI, Lower Limit 95% Confidence Interval; ULCI, Upper Limit 95% Confidence Interval. Bold numbers in the table indicated statistically significant at 0.05, which bootstrap interval did not include 0

Bootstrap analysis revealed that negative life events had an aggregate impact of 0.5994 (95% CI 0.312–0.8868), while the mediating effect of borderline traits was 0.1896 (95% CI 0.0764–0.3286). Importantly, the direct effect of negative life events on NSSI remained significant at 0.4178 (95% CI 0.1124–0.7232), indicating a partial mediation effect.

Similarly, we observed that both the total effect and direct effect of negative life events about interpersonal relationships and being punished for NSSI were significant, thereby supporting the presence and validity of the partial mediating role of borderline traits in the link between negative life events and nonsuicidal self-injury. The percentages of the mediating effects of borderline traits on total negative life events, interpersonal relationships, and being punished were 31.61%, 24.35%, and 25.11%, respectively.

Furthermore, we found that the influence of negative life events related to academic pressure, loss, and other factors on NSSI was fully mediated by borderline traits. Their total effects were significant, while the direct effects were not statistically significant. Finally, we did not observe statistically significant indirect effects of health-related adjustment on NSSI, indicating that borderline traits did not serve as a mediator in the connection between adverse life events and NSSI behaviours.

### Subgroup analysis

Because two-thirds of depressive patients with borderline traits exhibited suicidal behaviour before the age of 18, we stratified participants into two age groups, with and without participants aged 18 or older, to examine potential differences in the link between negative life events and nonsuicidal self-injury.

We observed that individuals with NSSI, regardless of age (over or under 18 years), were more likely to be female, have attempted suicide in the past (Supplemental Table 1), and display more severe clinical symptoms (Supplemental Table 2). Furthermore, the results of multivariate logistic regression analyses indicated that borderline traits remained a significant contributory factor for NSSI even after controlling for potential confounding variables (Supplemental Table 3).

Our research on the potential mediating function of borderline traits on the correlation between negative life events and nonsuicidal self-injury (NSSI) among minors and young adults is illustrated in Supplemental Figs. 2 and 3. Supplemental Tables 4 and 5 report more details. For those who were 18 years of age or younger, the influence of negative life events about academic pressure and being punished on NSSI was fully or partially mediated by borderline traits (BSL-23). Conversely, among individuals aged over 18 years, no statistically significant indirect effects of academic pressure on NSSI were reported, indicating that borderline traits did not act as a mediating factor in the relationship. However, negative life events related to being punished were fully mediated by borderline traits among individuals aged over 18 years.

## Discussion

Our recent study explored the connection between adverse life events and NSSI behaviours within a clinical cohort of depressed teenagers. Additionally, we delved into the potential role of borderline personality traits as mediators in this relationship. The most significant findings from this research suggest that an indirect correlation exists between negative life events during adolescence and nonsuicidal self-injury, and borderline personality traits mediate this connection, even when accounting for depressive symptoms. These results imply that youth who encounter a greater frequency of adverse life events could be more vulnerable to developing NSSI, and negative life events indirectly influence nonsuicidal self-injury through borderline personality traits. Depressed youth who were more vulnerable to NSSI behaviours often endured negative life events such as interpersonal relationships, academic pressure, being punished, and loss. For those under 18 years old, negative life events often involved academic pressure and being punished, while early-adult depressed patients tended to experience life events related to punishment.

The clinical sample collected for this study revealed a prevalence of NSSI of 59.47%, which is similar to previous research [37]. Furthermore, NSSI was more prevalent among female adolescents who had attempted suicide in the past and exhibited more severe clinical symptoms, which is also similar to previous results [38]. These outcomes corroborate earlier discoveries that established a robust correlation between NSSI and an elevated occurrence of suicidal thoughts, heightened suicide planning, and more suicide attempts [39].

Multivariate analyses were performed to assess the correlation between adverse life experiences and nonsuicidal self-injury. Even after controlling for depressive symptoms, our findings revealed that in young patients with NSSI, the connection between negative life events involving interpersonal relationships, borderline traits, and NSSI remained significant. This observation is in line with the results of prior investigations [40] and indicates that borderline traits play a prominent role in this context.

Moreover, when controlling for the patients’ depressive symptoms, our mediation analysis revealed that negative life events involving interpersonal relationships increased the propensity of depressed youth to engage in NSSI. Moreover, this relationship was mediated by borderline traits. As we all know, personality disorders (PDs) are considered to be the result of the interactions between temperamental vulnerabilities and adverse life events (stressors) [41], which is consistent with recent findings on gene/environment interaction in BPD [42]. Patients with borderline traits tend to experience interpersonal difficulties, including unstable relationships and a fear of abandonment [43]. Interpersonal hypersensitivity and emotional instability, which manifest as heightened reactions to negative interpersonal situations among youth, are central symptoms of BPD [44]. Impulsivity in BPD frequently manifests as eruptions within interpersonal relationships, whereas self-harming behaviours are commonly triggered by the perceived threat of separation or rejection within these connections. Youths may perceive self-harming as an extreme coping mechanism employed to regulate their intense negative emotions and prevent abandonment, which can lead to the formation of a detrimental cycle [11, 45].

Stress exposure models of psychopathology have demonstrated that individuals exposed to more life stressors tend to develop more negative psychological states [21]. When experiencing difficulties in emotional regulation, these individuals are more prone to employ NSSI as a mechanism for regulating their emotions [46, 47]. For adolescents, learning is a crucial developmental task that occupies a significant portion of their time [48]. This has a profound impact on them. Similarly, adolescents are more sensitive to the emotional experience of loss. Adolescents often face punitive events from parents and teachers due to their physical and psychological immaturity. These life stressors can make adolescents, especially those with depressive symptoms or borderline traits, more susceptible to emotional dysregulation, thus contributing to the occurrence of NSSI.

Furthermore, our results have implications for the management of NSSI among depressed youth, as they may not always respond adequately to standard pharmacological interventions, particularly in cases where there are comorbid borderline traits [10]. Major depressive disorder often co-occurs with BPD. In a comprehensive study, as many as 83% of individuals with BPD were found to have a lifetime prevalence of concurrent depression [7]. Accurately diagnosing this comorbidity can be clinically challenging due to the significant symptom overlap between depression and BPD.

The primary treatment approach for BPD involves psychotherapy [49], which includes dialectical behaviour therapy, psychodynamic treatment, and empathic treatment. These therapies have been shown to reduce depression levels. However, there is scant evidence supporting the efficacy of antidepressant medications in this context. Effective treatment of BPD frequently results in the remission of depression symptoms, whereas antidepressants typically offer only modest benefits for depression that co-occurs with BPD [44]. This underscores the importance of clinicians assessing BPD features in patients with depression who engage in NSSI [43]. This assessment may be particularly valuable in acute care settings where evaluating a patient’s long-term personality structure and comprehensive medical history may be challenging [43].

### Limitations and strengths

Compared to other studies, we primarily focused on depressed youth and quantitatively assessed their borderline personality traits. Our study elucidates the role of social factors and personality traits in NSSI among depressed youth. However, there are certain limitations associated with this study. First, it was a cross-sectional study, preventing us from establishing temporal priorities among the relationships in mediation analyses or examining causal relationships between negative life events, borderline personality traits and nonsuicidal self-injury. A prospective cohort study will be conducted in the future to investigate the causal relationships among these fctors. Second, self-report questionnaires were utilized to assess youths’ negative life events and borderline traits, which may have introduced recall bias.

## Conclusion

Our research provides therapeutic insights into the early identification and management of NSSI in depressed teenage patients and emphasizes the function of borderline traits as a mediator between negative life events and nonsuicidal self-injury. First, the significance of evaluating borderline personality traits in therapeutic settings is underscored by our results, particularly for depressed teenagers who have experienced negative events like interpersonal relationships, academic pressure, being punished, and loss. Second, the study suggested that psychotherapy targeting borderline traits may offer clinical benefits for depressed youth with NSSI.

### Electronic supplementary material

Below is the link to the electronic supplementary material.

Supplementary Material 1

## Data Availability

The datasets generated and/or analysed during the current study are not publicly available due to the nature of this research, participants of this study did not agree for their data to be shared publicly, but are available from the corresponding author on reasonable request.
