# Supplementary material for: Borderline personality traits mediate the relationship between negative life events and nonsuicidal self-injury in a clinical sample with youth depression
Source: BMC Psychiatry. 2024 May 16;24:370. doi: 10.1186/s12888-024-05821-0 (PMC11100148; doi:10.1186/s12888-024-05821-0)
Supplement: Supplementary file 1 — Supplementary Material 1 [file 12888_2024_5821_MOESM1_ESM.docx]

Supplemental Table1 clinical characteristic of the sample of depressive patients by interval of 18 years old.

|  | **No. (%)** | | | | | | | | | |
| --- | --- | --- | --- | --- | --- | --- | --- | --- | --- | --- |
|  | **age<=18** | | | **t/Z/X^2^** | **P value** | **age＞18** | | | **t/Z/X^2^** | **P value** |
|  | **Total**  **（n=149）** | **NSSI**  **(n=106)** | **Non-NSSI**  **(n=43)** |  |  | **Total**  **（n=189）** | **NSSI**  **(n=95)** | **Non-NSSI**  **(n=94)** |  |  |
| **Age (mean (SD))** | 16.07 ± 1.56 | 15.92 ± 1.60 | 16.47 ± 1.42 | 1.963 | .051 | 21.42 ± 2.04 | 21.18 ± 2.00 | 21.66 ± 2.06 | 1.629 | .105 |
| **Female** | 118 (79.19) | 90 (84.91) | 28 (65.12) | 7.271 | .007 | 134 (70.9) | 77 (81.05) | 57 (60.64) | 9.544 | .002 |
| **Education** |  |  |  | 6.263 | .086 |  |  |  | 1.978 | .557 |
| Middle school | 6 (4.0) | 2 (1.9) | 4 (9.3) |  |  | 4 (2.12) | 2 (2.11) | 2 (2.13) |  |  |
| Junior high school | 50 (33.6) | 40 (37.7) | 10 (23.3) |  |  | 10 (5.29) | 7 (7.37) | 3 (3.19) |  |  |
| Senior high School or secondary school | 82 (55.0) | 57 (53.8) | 25 (58.1) |  |  | 28 (14.81) | 15 (15.79) | 13 (13.83) |  |  |
| University or above | 11 (7.4) | 7 (6.6) | 4 (9.3) |  |  | 147 (77.78) | 71 (74.74) | 76 (80.85) |  |  |
| **Marriage** |  |  |  | 3.729 | .155 |  |  |  | 2.910 | .234 |
| Single | 109 (73.15) | 78 (73.58) | 31 (72.09) |  |  | 139 (73.54) | 65 (68.42) | 74 (78.72) |  |  |
| In love | 31 (20.81) | 24 (22.64) | 7 (16.28) |  |  | 44 (23.28) | 27 (28.42) | 17 (18.09) |  |  |
| Married | 9 (6.04) | 4 (3.77) | 5 (11.63) |  |  | 6 (3.17) | 3 (3.16) | 3 (3.19) |  |  |
| **Family income per month** |  |  |  | 0.342 | .952 |  |  |  | 10.413 | .015 |
| ＜3000 | 11 (7.38) | 7 (6.60) | 4 (9.30) |  |  | 10 (5.29) | 8 (8.42) | 2 (2.13) |  |  |
| 3000-5000 | 42 (28.19) | 30 (28.30) | 12 (27.91) |  |  | 48 (25.4) | 25 (26.32) | 23 (24.47) |  |  |
| 5000-10000 | 56 (37.58) | 40 (37.74) | 16 (37.21) |  |  | 53 (28.04) | 18 (18.95) | 35 (37.23) |  |  |
| ＞10000 | 40 (26.85) | 29 (27.36) | 11 (25.58) |  |  | 78 (41.27) | 44 (46.32) | 34 (36.17) |  |  |
| **History of suicide attempts** |  |  |  | 10.069 | .006 |  |  |  | 8.407 | .011 |
| None | 84 (56.4) | 51 (48.1) | 33 (76.7) |  |  | 122 (64.55) | 52 (54.74) | 70 (74.47) |  |  |
| 1-2 times | 58 (38.9) | 49 (46.2) | 9 (20.9) |  |  | 62 (32.8) | 39 (41.05) | 23 (24.47) |  |  |
| ≥3 times | 7 (4.7) | 6 (5.7) | 1 (2.3) |  |  | 5 (2.65) | 4 (4.21) | 1 (1.06) |  |  |

Supplemental Table2: Between-group differences in borderline traits, depression symptom and adolescent life events by interval of 18 years old.

| **Non-suicidal Self- injury** | **age<=18** | | |  |  | **age＞18** | | |  |  |
| --- | --- | --- | --- | --- | --- | --- | --- | --- | --- | --- |
|  | **Overall**  **（n=149）** | **NSSI**  **(n=106)** | **Non-NSSI**  **(n=43)** | **t/Z** | **P value** | **Total**  **(n = 189)** | **NSSI**  **(n = 95)** | **Non-NSSI**  **(n = 94)** | **t/Z** | **P value** |
| **Borderline traits (BSL-23)** |  |  |  |  |  |  |  |  |  |  |
| BSL-23 total, Mean ± SD | 48.68 ± 23.15 | 53.00 ± 21.05 | 38.02 ± 24.83 | -3.732 | <.001 | 40.79 ± 23.11 | 48.04 ± 22.35 | 33.46 ± 21.61 | -4.560 | <.001 |
| **Depression symptom( HAMD-17)** |  |  |  |  |  |  |  |  |  |  |
| Anxiety/somatization, Mean ± SD | 7.19 ± 3.48 | 7.30 ± 3.22 | 6.91 ± 4.09 | -0.566 | .573 | 6.23 ± 3.16 | 6.60 ± 2.84 | 5.86 ± 3.43 | -1.609 | .109 |
| Cognitive impairment, Mean ± SD | 4.32 ± 2.11 | 4.67 ± 2.04 | 3.44 ± 2.06 | -3.323 | .001 | 3.52 ± 2.21 | 4.08 ± 2.19 | 2.96 ± 2.10 | -3.609 | <.001 |
| Weight, M (Q₁, Q₃) | 0.00 (0.00 - 1.00) | 0.00 (0.00 - 1.00) | 0.00 (0.00 - 1.00) | -1.383 | .167 | 0.00 (0.00 - 1.00) | 0.00 (0.00 - 1.00) | 0.00 (0.00 - 1.00) | -0.955 | .340 |
| Hindrance, Mean ± SD | 5.12 ± 2.24 | 5.17 ± 2.16 | 5.00 ± 2.44 | -0.419 | .676 | 4.52 ± 2.06 | 4.79 ± 2.16 | 4.26 ± 1.93 | -1.789 | .075 |
| Sleep disturbance, Mean ± SD | 2.67 ± 1.88 | 2.80 ± 1.90 | 2.35 ± 1.81 | -1.334 | .184 | 2.58 ± 1.97 | 2.65 ± 2.02 | 2.51 ± 1.93 | -0.494 | .622 |
| HAMD-17 total, Mean ± SD | 19.82 ± 8.03 | 20.52 ± 7.59 | 18.09 ± 8.88 | -1.682 | .095 | 17.33 ± 7.94 | 18.58 ± 7.75 | 16.06 ± 7.97 | -2.200 | .029 |
| **Negative life events (ASLEC)** |  |  |  |  |  |  |  |  |  |  |
| Interpersonal relationship, Mean ± SD | 14.17 ± 4.71 | 15.46 ± 4.35 | 11.00 ± 4.04 | -5.786 | <.001 | 11.50 ± 4.38 | 12.29 ± 4.51 | 10.69 ± 4.10 | -2.555 | .011 |
| Academic pressure, Mean ± SD | 12.14 ± 4.11 | 12.66 ± 4.15 | 10.86 ± 3.77 | -2.460 | .015 | 10.84 ± 4.03 | 11.17 ± 4.30 | 10.50 ± 3.73 | -1.140 | .256 |
| Being punished, M (Q₁, Q₃) | 12.00 (10.00 - 16.00) | 13.00 (11.00 - 17.75) | 10.00 (8.00 - 12.00) | -4.630 | <.001 | 10.00 (8.00 - 14.00) | 11.00 (9.00 - 15.00) | 9.50 (8.00 - 12.00) | -2.595 | .009 |
| Loss, M (Q₁, Q₃) | 4.00 (3.00 - 6.00) | 4.00 (3.00 - 6.00) | 4.00 (3.00 - 5.00) | -1.401 | .161 | 4.00 (3.00 - 6.00) | 4.00 (3.00 - 7.00) | 3.00 (3.00 - 5.00) | -2.110 | .035 |
| Health-related adjustment, Mean ± SD | 8.38 ± 2.77 | 8.57 ± 2.88 | 7.91 ± 2.45 | -1.321 | .189 | 8.29 ± 2.69 | 8.72 ± 2.93 | 7.86 ± 2.36 | -2.204 | .029 |
| Others, Mean ± SD | 8.72 ± 2.99 | 9.20 ± 3.01 | 7.53 ± 2.61 | -3.173 | .002 | 7.75 ± 2.82 | 8.17 ± 2.83 | 7.32 ± 2.77 | -2.085 | .038 |
| ASLEC total, Mean ± SD | 60.47 ± 15.79 | 64.18 ± 15.57 | 51.33 ± 12.33 | -4.829 | <.001 | 53.83 ± 15.90 | 56.88 ± 16.72 | 50.73 ± 14.48 | -2.703 | .008 |

.Note. BSL-23 = 23‐Item Borderline Symptom List. HAMD-17 = the17-item Hamilton Depression Rating Scale (Anxiety/somatization, cognitive impairment, weight, hindrance and sleep disturbance are factors of HAMD-17). ASLEC = Adolescent Self-Rating Life Events CheckList (Interpersonal relationship, academic pressure, being punished, loss, health-related adjustment, and others are factors of ASLEC ).

Supplemental Table3 Multivariate logistic regression analyses examining predictors of NSSI by interval of 18 years old.

| **Multivariate analysis** | **age<=18** | | | | **age＞18** | | | |
| --- | --- | --- | --- | --- | --- | --- | --- | --- |
|  | **OR** | **95%CI** | **P value** | **P value adjusted** | **OR** | **95%CI** | **P value** | **P value adjusted** |
| **Sex** |  |  |  |  |  |  |  |  |
| Female | 2.019 | 0.711-5.736 | .187 | 1.000 | 3.077 | 1.429-6.626 | .004 | 0.064 |
| **History of suicide attempts** |  |  | .024 | 0.288 |  |  | .241 | 1.000 |
| 1-2 times | 4.295 | 1.507-12.239 | .006 | 0.072 | 1.691 | 0.823-3.475 | .153 | 1.000 |
| ≥3 times | 2.000 | 0.124-32.307 | .625 | 1.000 | 3.938 | 0.297-52.236 | .299 | 1.000 |
| **Income per month** |  |  |  |  |  |  | .06 | 0.960 |
| 3000-5000 |  |  |  |  | 0.487 | 0.083-2.848 | .424 | 1.000 |
| 5000-10000 |  |  |  |  | 0.191 | 0.033-1.103 | .064 | 1.000 |
| ＞10000 |  |  |  |  | 0.490 | 0.088-2.736 | .416 | 1.000 |
| **BSL-23** | 1.028 | 1.001-1.056 | .041 | 0.492 | 1.030 | 1.010-1.051 | .003 | 0.048 |
| **HAMD-17** | 0.977 | 0.904-1.055 | .549 | 1.000 | 0.988 | 0.939-1.040 | .644 | 1.000 |
| **ASLEC** |  |  |  |  |  |  |  |  |
| Interpersonal relationship | 1.291 | 1.112-1.498 | .001 | 0.012 | 1.051 | 0.946-1.167 | .352 | 1.000 |
| Academic pressure | 0.966 | 0.839-1.112 | .631 | 1.000 | 0.945 | 0.848-1.053 | .307 | 1.000 |
| Being punished | 1.186 | 1.012-1.390 | .036 | 0.432 | 1.043 | 0.918-1.185 | .519 | 1.000 |
| Loss | 1.090 | 0.891-1.333 | .401 | 1.000 | 1.096 | 0.942-1.275 | .235 | 1.000 |
| Health-related adjustment | 0.731 | 0.555-0.963 | .026 | 0.312 | 1.030 | 0.863-1.229 | .746 | 1.000 |
| Others | 0.974 | 0.774-1.226 | .825 | 1.000 | 0.886 | 0.741-1.059 | .184 | 1.000 |

Note. OR = Odds Ratio. BSL-23 = 23‐Item Borderline Symptom List. HAMD-17 = the17-item Hamilton Depression Rating Scale. ASLEC = Adolescent Self-Rating Life Events CheckList.

P value adjusted: using the Bonferroni correction

Supplemental Table4: Results of mediating effect for borderline traits between adolescent negative life events and NNSI among depressed adolescents who age<=18.

| Paths | **Bootstrap effects** | | | | | |
| --- | --- | --- | --- | --- | --- | --- |
|  | **Total effect** | **LLCI** | **ULCI** | **Indirect effect** | **LLCI** | **ULCI** |
| Total negative life events →Borderline traits→non-suicidal self-injury | 1.0851 | **0.5377** | **1.6326** | 0.1835 | -0.016 | 0.4724 |
| Interpersonal relationship→Borderline traits→non-suicidal self-injury | 1.2543 | **0.7137** | **1.7949** | 0.1692 | -0.0501 | 0.4387 |
| Academic pressure→Borderline traits→non-suicidal self-injury | 0.4684 | **0.0519** | **0.885** | 0.1221 | **0.0070** | **0.3532** |
| Being punished→Borderline traits→non-suicidal self-injury | 1.0117 | **0.4665** | **1.5568** | 0.1093 | **0.0108** | **0.3011** |
| Loss→Borderline traits→non-suicidal self-injury | 0.2218 | -0.1883 | 0.6318 | 0.1336 | **0.0231** | **0.3465** |
| Health-related adjustment→Borderline traits→non-suicidal self-injury | 0.1284 | -0.3346 | 0.5913 | 0.1753 | **0.0351** | **0.4205** |
| Others→Borderline traits→non-suicidal self-injury | 0.4783 | **0.0364** | **0.9202** | 0.1924 | **0.0347** | **0.4710** |

LLCI, Lower Limit 95% Confidence Interval; ULCI, Upper Limit 95% Confidence Interval. Bold numbers in the table indicated statistically significant at 0.05, which bootstrap interval did not include 0.

Supplemental Table5: Results of mediating effect for borderline traits between adolescent negative life events and NNSI among depressed adolescents who age＞18.

| Paths | **Bootstrap effects** | | | | | |
| --- | --- | --- | --- | --- | --- | --- |
|  | **Total effect** | **LLCI** | **ULCI** | **Indirect effect** | **LLCI** | **ULCI** |
| Total negative life events →Borderline traits→non-suicidal self-injury | 0.3326 | **0.0027** | **0.6624** | 0.2135 | **0.0748** | **0.4186** |
| Interpersonal relationship→Borderline traits→non-suicidal self-injury | 0.3091 | -0.0084 | 0.6265 | 0.1795 | **0.0672** | **0.3416** |
| Academic pressure→Borderline traits→non-suicidal self-injury | 0.048 | -0.2576 | 0.3535 | 0.1093 | **0.0137** | **0.2578** |
| Being punished→Borderline traits→non-suicidal self-injury | 0.3486 | **0.0253** | **0.6719** | 0.1939 | **0.069** | **0.3795** |
| Loss→Borderline traits→non-suicidal self-injury | 0.3259 | **0.0044** | **0.6473** | 0.0666 | -0.0298 | 0.2026 |
| Health-related adjustment→Borderline traits→non-suicidal self-injury | 0.2836 | -0.0498 | 0.617 | 0.1460 | **0.0391** | **0.3263** |
| Others→Borderline traits→non-suicidal self-injury | 0.238 | -0.0794 | 0.5554 | 0.3138 | **0.1174** | **0.5502** |

LLCI, Lower Limit 95% Confidence Interval; ULCI, Upper Limit 95% Confidence Interval. Bold numbers in the table indicated statistically significant at 0.05, which bootstrap interval did not include 0.





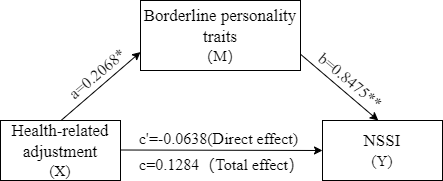

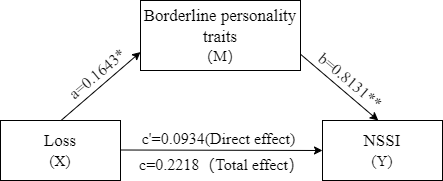

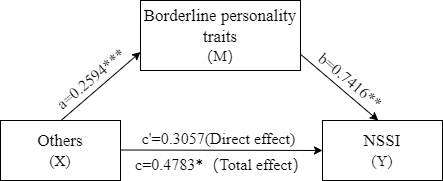


a

b

c

d

e

f


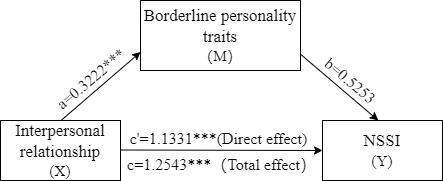

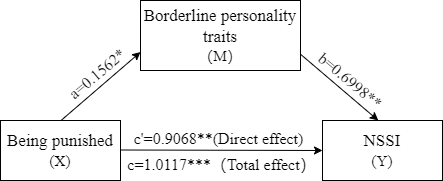

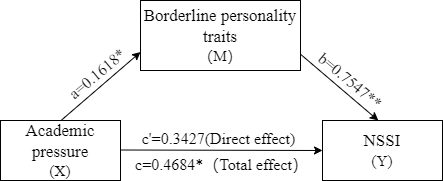


Supplemental Figure1 . Borderline traits partially mediate the relationship between negative life events about: (a) interpersonal relationship, (c) being punished, and borderline personality traits fully explain the association between adolescent life events about: (b) academic pressure, (d) loss, and (f) others and the likelihood of participating in NSSI behaviours. (adjusting for gender, previous suicidal history and depressive symptom). * p < .05; ** p < .01; *** p < .001.





Supplemet Figure 2 . For depressed adolescents <=18 years old, borderline traits partially mediate the relationship between adolescent life events about: (c) being punished and borderline traits fully mediate the relationship between adolescent life events about: (b) academic pressure, (f)others, and the probability of engaging in non-suicidal self-injury (controlling for previous suicidal history and depressive symptom). * *p* < .05; ** *p* < .01; *** *p* < .001.





Supplemet Figure 3 . For depressed adolescents ＞18 years old, borderline traits fully mediate the relationship between adolescent life events about: (c) being punished and the probability of engaging in non-suicidal self-injury (controlling for gender and depressive symptom). * p < .05; ** p < .01; *** p < .001.
